# Simultaneous detection and identification of Peste des petits ruminants Virus Lineages II and IV by MCA-Based real-time quantitative RT-PCR assay within single reaction

**DOI:** 10.1186/s12917-023-03568-6

**Published:** 2023-01-16

**Authors:** Jingyu Tang, Hanyu Du, Aoxing Tang, Nannan Jia, Jie Zhu, Chuanfeng Li, Chunchun Meng, Guangqing Liu

**Affiliations:** Shanghai Veterinary Research Institute, Chinese Academy of Agricultural Sciences, 200241 Shanghai, PR China

**Keywords:** Peste des petits ruminants, Real-time qRT-PCR, Melting curve analyses, PPRV lineage II, PPRV lineage IV

## Abstract

**Background:**

Peste des petits ruminants (PPR) disease is a cross-species infectious disease that severely affects small ruminants and causes great losses to livestock industries in various countries. Distinguishing vaccine-immunized animals from naturally infected animals is an important prerequisite for the eradication of PPR. At present PPRV are classified into lineages I through IV, and only one vaccination strain, Nigeria/75/1, belongs to lineage II, but all of the epidemic strains in China at present are from lineage IV.

**Results:**

To achieve this goal, we developed an SYBR Green I real-time qRT-PCR method for rapid detection and identification of PPRV lineages II and IV by analyzing different melting curve analyses. The negative amplification of other commonly circulating viruses such as orf virus, goat poxvirus, and foot-and-mouth disease virus demonstrated that primers targeting the L gene of PPRV were extremely specific. The sensitivity of the assay was assessed based on plasmid DNA and the detection limit achieved was 100 copies of PPRV lineages II and IV.

**Conclusion:**

Since the method has high sensitivity, specificity, and reproducibility, it will be effectively differentiated PPRV lineages II from PPRV lineages IV in PPRV infected animals.

**Supplementary Information:**

The online version contains supplementary material available at 10.1186/s12917-023-03568-6.

## Background

Peste des petits ruminants (PPR) is a highly contagious, cross-species disease caused by the Peste des petits ruminants virus (PPRV) [1, 2]. It can infect not only small ruminants such as goats and sheep, but also large ruminants such as camels, buffalo, and wild animals [3, 4]. More than 68% of sheep and goats and 2.5 billion small ruminants reside in countries affected with PPRV, according to FAO data from 2018 [5]. The widespread distribution and prevalence of PPR have an important impact on economic trade and biodiversity.

PPRV belongs to the genus *Morbillivirus* of the family *Paramyxoviridae* [6]. According to the sequences of the N gene and F gene, PPRV can be classified into four lineages, I, II, III, and IV [7]. Lineages I and II are predominantly endemic in West Africa [8, 9], while lineage III is mainly found in the Arab region, and has also been reported in East Africa [10, 11], Lineage IV is mainly prevalent in the Middle East and Asia, and has also been reported in Africa [8,12,13]. PPR was first reported in the Tibet region in 2007 [14], then re-emerged in Xinjiang Uyghur Autonomous Region in 2013, and then spread to 23 provinces, autonomous regions, and municipalities of China [15,16]. China has adopted strict compulsory immunization and culling policies, which have effectively controlled the occurrence of the PPR. Currently, the live vaccine named the Nigeria/75/1 is the only vaccine widely used for the prevention of PPR in China. It can provide strong immune protection in goats and sheep, as it can proliferate continuously after injection. A major obstacle to eliminating PPR is the inability to distinguish vaccine groups from PPRV antibodies or antigen-positive animals in clinical settings. So, for the prevention and elimination of PPR, there is a great need for a simple assay that can detect and distinguish the PPRV vaccinated and infected groups at the same time.

Since the Nigeria/75/1 strain PPRV belongs to lineage II, while the PPRV prevalent in China all belong to lineage IV [15], and SYBR Green I real-time qRT-PCR assay was developed by analyzing all the published PPRV sequences of lineages II and IV. We use only one pair of primers that can detect all the lineages II and IV PPRV, and by analyzing different melting curve analyses (MCA), it can differentiate them conveniently and quickly. The parameters were optimized, and the sensitivity, specificity and reproducibility of the assay were assessed and compared with the traditional methods. The diagnostic application of the assay was carefully evaluated based on standard plasmid which cloned from the L gene of PPRV genome.

## Results

### Standard curve for the real-time qRT-PCR

**Fig. 1:**
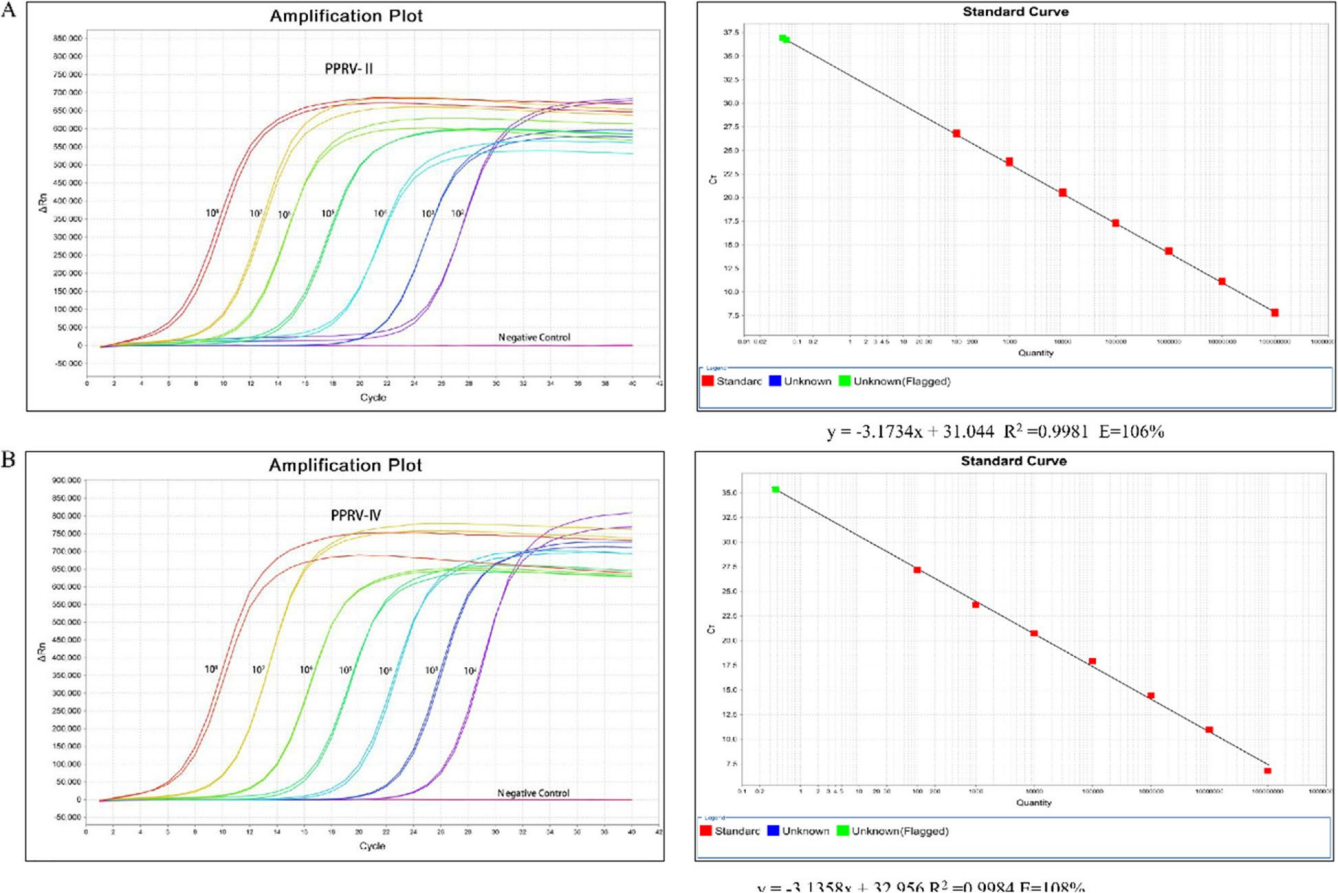
Amplification curves and standard curves of the real-time qRT-PCR assay to measure viral copy number of PPRV lineages II and IV. The tenfold diluted standard plasmid of PPRV lineages II and IV was tested with the same pair of primers. Regression lines between the Ct values and the input concentrations of PPRV lineage II (**A**) and PPRV lineage IV (**B**) plasmid DNA using SYBR Green I real-time qRT-PCR

Two linear standard curves were constructed using tenfold dilutions of PPRV lineages II and IV standard plasmids, using a dilution concentration of 1.0 × 10^8^-1.0 × 10^2^ copies/µL. All samples were tested in triplicate and independently. The equations for the PPRV lineages II and IV standard curves were y = -3.1734x + 31.044 and y = -3.1358x + 32.956, respectively. Standard curves analysis demonstrated that when the copy numbers were in the range of 10^8^ and 10^2^ copies/µL, the amplification efficiency of PPRV lineages II and IV was 106% and 108%, respectively. The correlation coefficient R^2^ value for the linear regression equation of PPRV lineages II and IV was 0.9981 and 0.9984 (Fig. 1A and B).

**Fig. 2:**
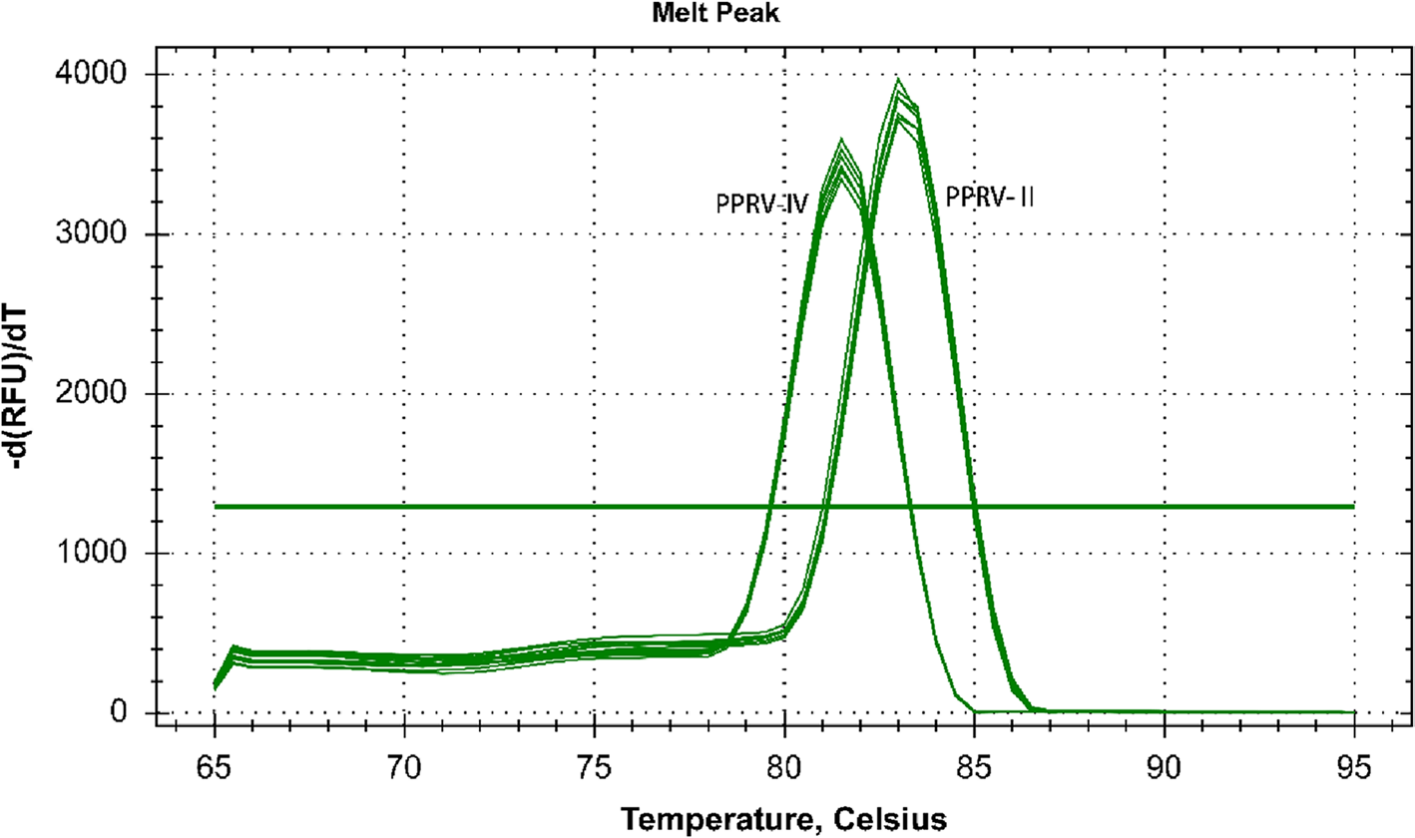
Melting curves of the real-time qRT-PCR assay to detect PPRV lineages II and IV. Amplification melting curve and melting temperature values (Tm) after SYBR Green I real-time qRT-PCR followed by melting curve analysis of PPRV lineages II and IV

In addition, the melting curves for both lineages II and IV were single peaks and melting temperatures (Tm) were 83.39℃ and 81.92℃, respectively (Fig. 2).

### Specificity, sensitivity and reproducibility of real-time qRT-PCR assay

**Fig. 3:**
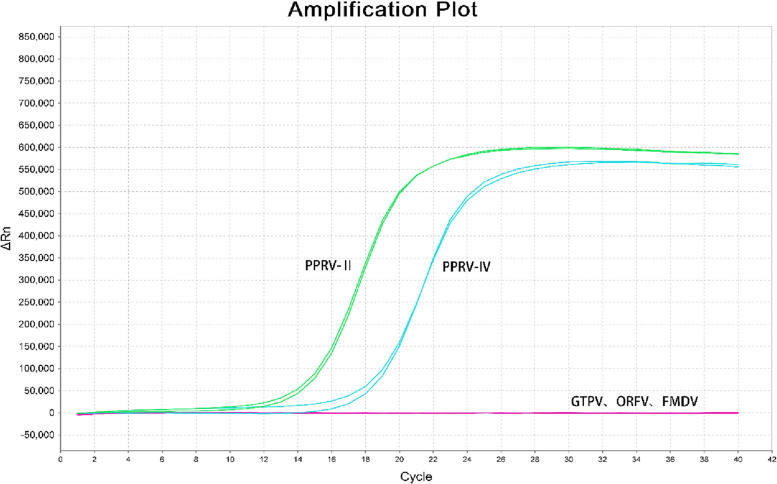
Specificity of the real-time qRT-PCR assay. The specific fluorescent signals were detected from cDNA of PPRV lineage II and PPRV lineage IV plasmids, and the dissociation curves showed that there were two specific product peaks for PPRV lineages II and IV, respectively, but no specific amplification for negative control (ORFV, GTPV, and FMDV)

To verify the specificity of the assay, lineage II PPRV, ORFV, GTPV, and FMDV genomic cDNA or DNA, and lineage IV PPRV plasmids were used as templates and amplified using the conditions described above. Only PPRV (lineages II and IV) was amplified in these samples and no specific peaks appeared in the other samples (Fig. 3), which indicated that the established real-time qRT-PCR assay was specific for PPRV and does not cross-react with other pathogens. The detection limits for PPRV lineages II and IV were 100 copies/µL.

Standard plasmids (PPRV lineages II and IV) were diluted (10^7^,10^5^,10^3^ copies/µL) to assess intra- and inter-assay reproducibility. And the CV of CT values were all less than 5%. Specifically, for PPRV lineage II plasmids, the CV of the inter- and intra- assays ranged from 0.06% to 0.49% and 0.07% to 6.09%, and for PPRV lineage IV plasmids, the CVs ranged from 0.06% to 0.30% and 1.03% to 6.49%, respectively (Table 1). These results indicate that the real-time qRT-PCR assay had good reproducibility.

**Table 1:** Variance analysis of Ct values for real-time qRT-PCR assay

DNA standard (copies/µL)	Intra-assay			Inter-assay		
	Mean (Ct)	SD	CV(%)	Mean (Ct)	SD	CV(%)
PPRV-II						
10^7^	10.09	0.050	0.49	10.55	0.643	6.09
10^5^	16.45	0.052	0.31	15.89	0.793	4.99
10^3^	23.56	0.016	0.06	23.58	0.017	0.07
PPRV-IV						
10^7^	10.41	0.007	0.06	9.94	0.638	6.42
10^5^	17.11	0.052	0.30	17.00	0.177	1.03
10^3^	25.45	0.002	0.01	24.33	1.581	6.49

### Detection and identification of PPRV in test samples

To evaluate the assay’s potential performance in the clinical setting, we calculate the melting temperature in the real-time qRT-PCR target region of all the PPRV lineage II and IV strains (9 lineage II strains, 25 lineage IV strains isolated from foreign countries, and 37 lineage IV strains from China) (Table 2). The average, median, and standard deviation melting temperature of lineage II (IV) amplified area are 82.34℃ (80.97℃), 82.30 ℃ (81 ℃), and 0.19℃ (0.14℃), respectively. Furthermore, there was no significant difference between lineage IV strains isolated from China and foreign countries. The DNA denaturation temperature diversity between the two lineages is about 1.3℃ which can be easily reflected after MCA, it indicates this assay can efficiently detect and differentiate Nigeria/75/1 and PPRV lineage IV isolates when used in the future.

**Fig. 4:**
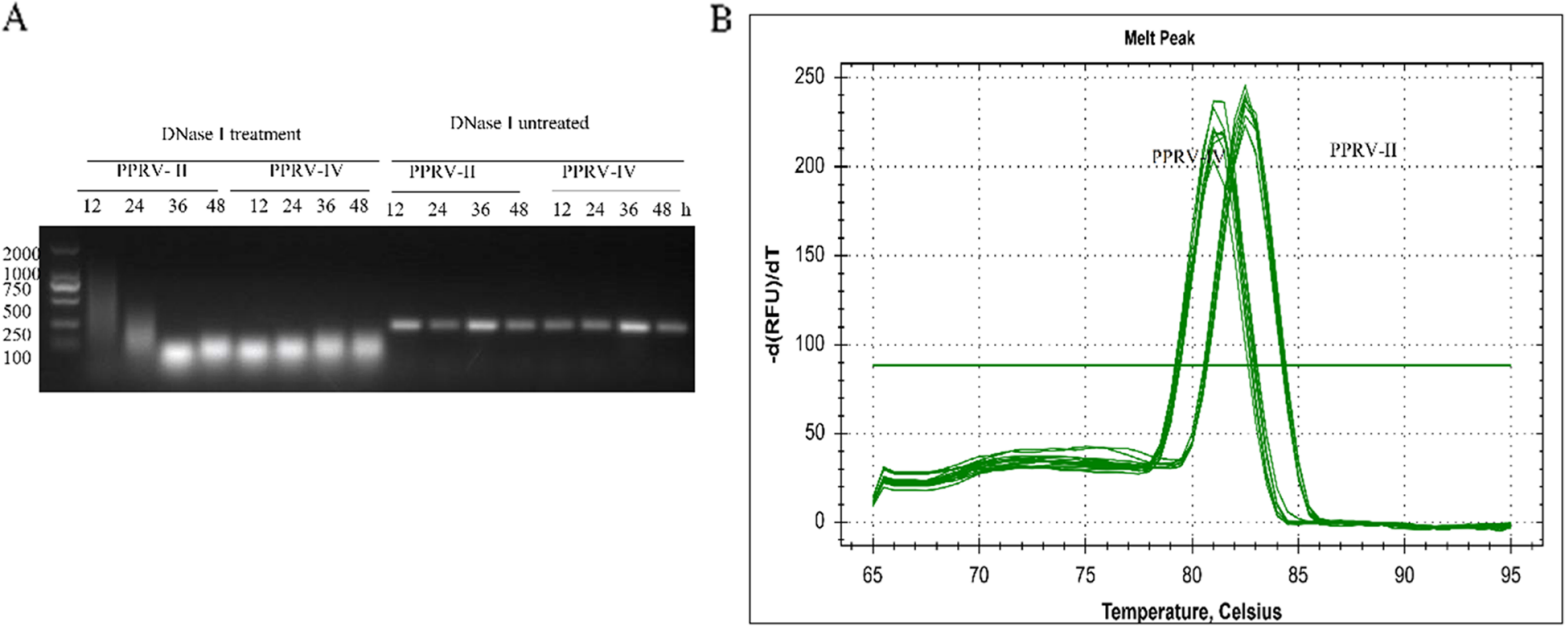
Detection and identification of PPRV in test samples. **A** Total RNA of PPRV-L gene transfected cell samples was digested with DNase I. HEK-293T cells were transfected with lineages II and IV PPRV-L gene plasmids. Cell samples were collected at 12, 24, 36 and 48 h after transfection. Total RNA was extracted and digested with DNase I. **B** PPRV lineages II and IV were detected and differentiated in cell samples by the real-time qRT-PCR assay. After the RNA of PPRV-L gene transfected cell samples was completely digested by DNase I, it was reversed transcribed and detected by the established real-time qRT-PCR assay

To evaluate the application of the method, total RNA from 16 transfected cell samples was extracted and reverse transcribed after digested with DNase I (Fig. 4A), and then detected by the established assay. The results showed that the method was able to distinguish the PPRV lineages II and IV genomic RNA clearly (Fig. 4B). Since the detection results of standard plasmids and RNA samples are highly consistent, it is suggested that this method can effectively amplify and distinguish clinical samples containing PPRV lineages II and IV.

**Table 2:** Calculation the melting temperature in the qRT-PCR amplified region of all the PPRV lineages II and IV strains

Foreign Strains (Lineage II)	Accession no.	Melting Temp. ℃	Foreign Strains (Lineage IV)	Accession no.	Melting Temp. ℃	Chinese Strains (Lineage IV)	Accession no.	Melting Temp. ℃	Chinese Strains (Lineage IV)	Accession no.	Melting Temp. ℃
Nigeria/75/1	X74443	82.49	Izatnagar/94	KR140086	81.19	China/Tibet/0701	EU360596	80.82	CH/HNZK/2014	KM089831	80.82
Nigeria/75/1cells	HQ197753	82.49	Sungri/96	KF727981	81.19	China/Tib/07	JF939201	80.82	ChinaSC2014	MF443338	81.0
Sierra Leone/048/2011	MF741712	82.3	Sungri 1996 MSD	KJ867542	81.19	China/33/2007	KX421388	80.82	ChinaNX2014	MF443340	81.0
CIV/01/P/2009	KR781451	82.67	Israel-2536/Hebron/1997	OL310687	81.38	China/Tibet/Bharal/2008	JX217850	80.82	ChinaLN2014	MF443341	81.0
GhanaNK1 2010	KJ466104	82.49	Turkey 2000	NC-006383	81.19	China/XJ4/2013	KX421386	81.0	ChinaJS2014	MF443343	81.0
Benin/B1/1969	KR781450	82.12	Bangladesh/BD2/2008	MG581412	80.82	China/XJ5/2013	KX421387	81.0	ChinaHeN2014	MF443347	81.0
Lib/2015	KU236379	82.30	Morocco 2008	KC594074	81.19	China/XJYL/2013	KM091959	81.0	ChinaGZ2014	MF443349	81.0
Benin/10/2011	KR781449	82.12	Ethiopia 2010	KJ867541	81.0	China/XJ3/2013	KX421385	81.0	ChinaGS2014	MF443351	81.0
SnDk11I13	KM212177	82.12	Kurdistan/2011	MK408669	81.19	China/XJ2/2013	KX421384	81.0	ChinaGD2014	MF443352	81.0
			DRC/Tshela/27/2012	OL310685	80.82	ChinaAH2014	MF443354	81.0	ChinaYN2014	MF443336	81.0
			NGYO2013-2162	KR828813	80.82	ChinaHB2014	MF443348	81.0	ChinaSX2014	MF443337	81.0
			India TN Gingee 2014	KR261605	80.82	ChinaCQ2014	MF443353	81.0	ChinaZJ2014	MF443335	81.0
			Israel/1571/Um-El-Fahem/2014	OL310704	81.19	ChinaHN2014	MF443345	81.0			
			IND/TN/GIN/2014/01	KT270355	80.82	ChinaJX2014	MF443342	81.0			
			IND/TN/VM/2014/02	KT860063	80.82	CH/HNZM/2014	KM089832	80.82			
			IND/TN/VEL/2015/03	KT860064	80.63	ChinaJL2014	MF443344	81.0			
			IND/TN/ED/2015/04	KT860065	80.63	ChinaSaX2014	MF443339	81.0			
			S15	KY885100	81.19	ChinaHLJ2014	MF443346	81.0			
			Georgia/Tbilisi/2016	MF737202	80.82	CH/HNNY/2014	KM089830	81.0			
			PPRV/Mongolia/9/2016	KY888168	80.82	ChinaGX2014	MF443350	81.0			
			IND/Delhi/2016/05	KX033350	80.82	GZL-14	KM816619	81.0			
			1008	MF678816	80.82	CH/GDDG/2014	KP868655	81.0			
			Siberian-ibex/Mongolia/2017-01	MZ061721	81.0	China/BJ/2014	KP260624	81.0			
			Turkey/Central-Anatolia/2018	MN657232	81.0	China/XJBZ/2015	KT633939	81.0			
			Mongolia/2017-01	MZ061722	81.0	PPRV-FY	KX354359	81.0

## Discussion

Since the emergence of PPRV in 1942, it has been widespread around the world, causing huge economic losses to the world’s animal husbandry. After the first report of PPRV in China in 2007, the outbreak of PPR still occurred, even though China adopted strict compulsory immunization and culling policies. At present, the vaccine strain is lineage II PPRV, but the dominant circulating lineage in China is IV. It is particularly important to detect and distinguish vaccine strain from wild strains.

At present, there are many detection methods for PPRV, which are mainly designed to detect all PPRV strains and can not distinguish between field PPRV strains and vaccine strains [17–19]. The real-time RT-PCR method designed by Li et al. [20] could specifically detect lineage IV PPRV, while the other three lineages could not be detected which limits of detection were found to be 100 copies/reaction. Yang established two detection methods for PPRV, real-time and lateral flow strip RT-RPA. The sensitivity of both was as low as 100 copies/reaction at 40℃ and as low as 150 copies/reaction at 39 ℃, respectively [21]. Several quantitative methods have been used to detect PPRV [22, 23], all of these methods can detect PPRV rapidly and effectively, but cannot distinguish between the PPRV vaccine strain and the prevalent strain in China.

The stability of the DNA duplexes can be affected by the GC content and length of the PCR products. Thus, the fluorescence signal of the SYBR Green I was different and could be used to specifically identify PCR products by Melting curve analysis (MCA). MCA in conjunction with real-time PCR, including dsDNA dye SYBR Green I and TaqMan probe-based types, has been widely used in previous studies including and single-nucleotide polymorphism(SNP)analysis [24–26]. The advantage of probe-based MCA is its ability for multiplex detection using a probe with specific melting temperatures. Compared with TaqMan probe-based MCA, SYBR Green I is a simple, rapid, and inexpensive method for testing. In our laboratory, PCR coupled with MCA has also been developed to detect and genotype duck hepatitis A virus types 1 and 3 [27]. In this study, we describe for the first time that only uses one pair of primers to distinguish lineage II and IV of PPRV based on the MCA and without reducing sensitivity. The sensitivity of the method is 100 copies/reaction. In addition, it does not cross-react with other viruses such as ORFV, GTPV and FMDV.

In order to evaluate the potential performance of the method in clinical application, first, the qRT-PCR target melting temperature of all PPRV lineages II and IV strains were calculated, and found the mean, median and standard deviation of the melting temperature of PPRV lineages II and IV amplified regions were significant differences, which were 82.34 °C (80.97 °C), 82.30 °C (81 °C) and 0.19 °C (0.14 °C), respectively. The denaturation temperature difference between PPRV lineages II and IV is about 1.3 °C, which can be easily distinguished by the melting curve. Then cell samples transfected with PPRV lineages II and IV plasmids were detected by the method and found that this method can effectively distinguish the two lineages of PPRV. In summary, the method can effectively detect and differentiate IV isolates of Nigeria/75/1 and PPRV families and has clinical application value.

## Conclusion

In this study, we developed an MCA-based SYBR Green I RT-PCR assay to effectively detect and differentiate PPRV lineages II and IV. The detection method is accurate, rapid, sensitive, reproducible, and easy to perform. Therefore, this detection method can be used for subsequent epidemiological investigations and PPR eradication.

## Methods

### Virus strains

Lineage II PPRV strain PPRV Nigeria/75/1 (GenBank accession no. HQ197753), which was a vaccine strain and propagated in Vero-SLAM cells. Lineage IV PPRV strain PPRV-FY (GenBank accession no. KX354359) was isolated and preserved in our lab. Orf virus (ORFV) strain AH-FD/2016/China, which was preserved in Shanghai Veterinary Research Institute (SHVRI), Chinese academy of agricultural science. The goat poxvirus (GTPV) strain AV41 and the foot-and-mouth disease virus (FMDV) strain O/Mya98/BY/2010 both were vaccine strains.

### Primer design

71 published whole-genome sequences (9 form lineage II PPRV and 62 form lineage IV PPRV, including 37 were Chinese strains and 25 were foreign strains) were retrieved from the GenBank database (Table 1). DNAstar software was used for comparison, and the L gene was used to design primers to detect and differentiate lineages II and IV PPRV, based on the principle that GC content difference in the inner region of two lineages PPRV gene sequences can produce different melting curves. The optimized forward primer F was 5’-ACAGGTTCGACAACATTCAAGCCA-3’ and reverse primer R was 5’-GCGAAGGTAGGTCAGAGAGCA-3’, and an expected 221 bp amplification product was generated.

### Standard plasmids template preparation

Lineage II PPRV genomic RNA was extracted from the viral suspension with RNeasy (Qiagen, Germany), and used immediately for cDNA synthesis. cDNA synthesis was performed with SuperScript II reverse transcriptase (RT) (Invitrogen, USA) and specific RT primers (R). The cDNA fragment of lineage II PPRV and synthesized plasmid of lineage IV PPRV were amplified with primer F and R. The 221 bp products were cloned into the pMD19-T vector (TaKaRa) and sequenced by Sangon (Shanghai, China).

### SYBR Green I-based quantitative real-time RT-PCR assay

The SYBR Green I-based real-time qRT-PCR was performed using Novozymes Ultra SYBR Mixture (Vazyme). Amplification was performed in a 20 µL reaction mixture containing 10 µL SYBR qPCR master mix (2×), 0.4 µL of each forward and reverse primers (10𝜇M), 2 𝜇L plasmid, and 7.2 µL H_2_O. The amplification conditions were 95 °C for 30 s, followed by 40 cycles of 95 °C for 5 s and 60 °C for 30 s. Two standard curves were generated with 10-fold serially diluted plasmids standards of 10^2^-10^8^ copies/µL. Each process was performed three times.

### Specificity, sensitivity and reproducibility of the real-time qRT-PCR assay

To determine the specificity of the method, PPRV lineage II, ORFV, GTPV and FMDV, genomic cDNA or DNA, and PPRV lineage IV plasmids were used as templates in real-time qRT-PCR according to the aforementioned reaction conditions.

To determine the sensitivity of the method, ten 10-fold serial dilutions of recombinant plasmids with concentrations from 10^8^ to 10^2^ copies/µL were used as templates. Each experiment was performed three times.

Standard plasmids were chosen to evaluate the reproducibility of the method. Every dilution was evaluated for intra-assay and inter-assay variation. Each process was tested three times in parallel under the same amplification conditions. Real-time qRT-PCR was used to detect the coefficient of variation (CV) and to determine the repeatability of the inter-batch detection.

### Evaluation of the real-time qRT-PCR assay

To evaluate the application of the method, lineages II and IV PPRV-L gene eukaryotic expression plasmids were constructed to simulate viral infection. HEK-293T cells were transfected with the lineages II and IV PPRV-L gene plasmids, then were collected at 12, 24, 36, and 48 h after transfection. Nucleic acids were extracted from these samples and tested by the real-time qRT-PCR.

## Supplementary Information

**Additional file 1.**

## Data Availability

The data presented in this manuscript are available through the corresponding author upon reasonable request. The datasets analyzed in the study are available in the NCBI GenBank database, [lineage II PPRV Nigeria/75/1 (https://www.ncbi.nlm.nih.gov/nuccore/HQ197753), lineage IV PPRV-FY (https://www.ncbi.nlm.nih.gov/nuccore/KX354359)].

## Abbreviations

PPR: Peste des petits ruminants
PPRV: Peste des petits ruminants virus
qRT-PCR: Quantitative reverse transcription polymerase chain reaction
FAO: Food and Agriculture Organization
ORFV: Orf virus
GTPV: Goat poxvirus
FMDV: Foot-and-mouth disease virus
MCA: Melting curve analyses
Ct: Cycle threshold
CV: Coefficients of variation
Tm: Melting temperatures
HRM: High-resolution melting
