# Supplementary figures and images for: Simultaneous detection and identification of Peste des petits ruminants Virus Lineages II and IV by MCA-Based real-time quantitative RT-PCR assay within single reaction
Source: BMC Vet Res. 2023 Jan 16;19:11. doi: 10.1186/s12917-023-03568-6 (PMC9841696; doi:10.1186/s12917-023-03568-6)

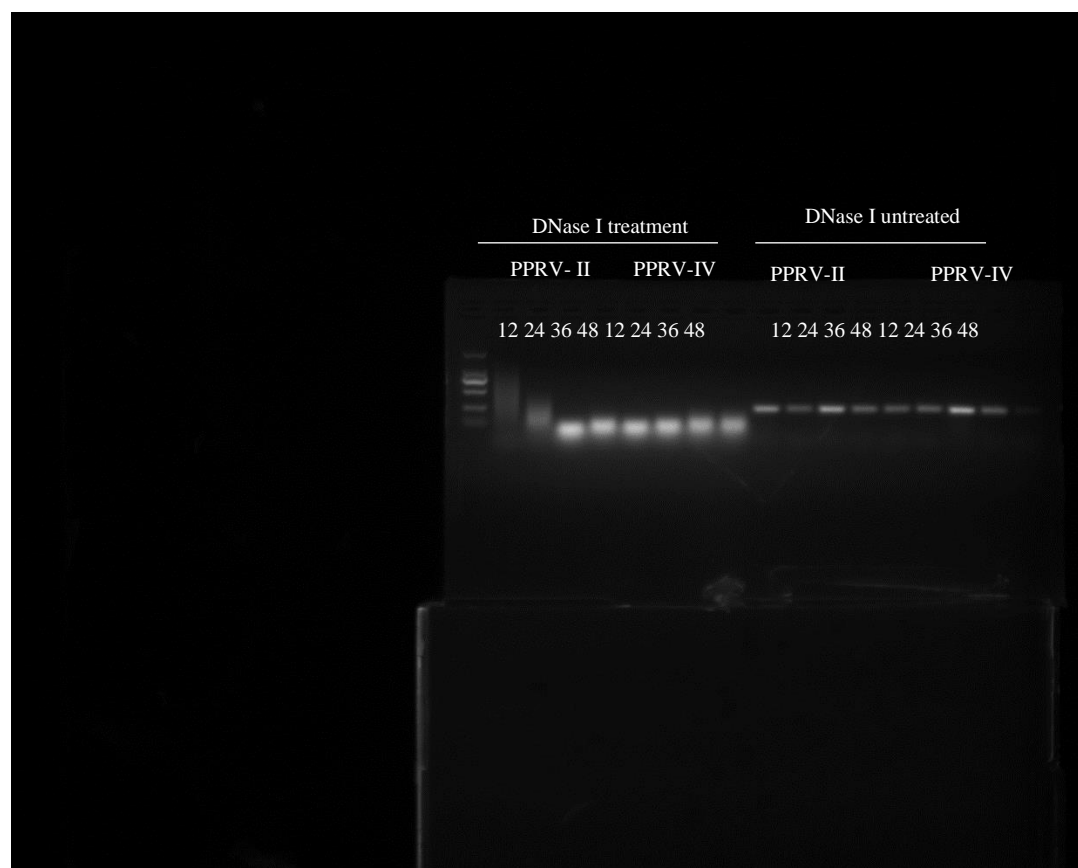

Supplement: Supplementary file 1 — Additional file 1. [file 12917_2023_3568_MOESM1_ESM.pdf]
